# Genomics and physiological characterizations of an acidotolerant nitrite-oxidizing *Nitrospira* enriched from freshwater pond

**DOI:** 10.1128/aem.01522-25

**Published:** 2025-09-18

**Authors:** Minji Kim, Yoichi Kamagata, Soo-Je Park

**Affiliations:** 1 Department of Biology, Jeju National University 34926, Jeju, South Korea; 2 Molecular Biosystem Research Institute, National Institute of Advanced Industrial Science and Technology (AIST) 538547 , Tsukuba, Ibaraki, Japan; Georgia Institute of Technology, Atlanta, Georgia, USA

**Keywords:** *Nitrospira*, nitrite oxidation, kinetics, acid-tolerant, genome, physiology

## Abstract

Nitrite-oxidizing bacteria (NOB) play a crucial role in global nitrogen cycling, yet their presence and adaptations in acidic environments remain poorly understood. This study unveils the cultivation and characterization of a novel acid-tolerant NOB, NS4 culture, affiliated with lineage II (*Nitrospira*_*D*) within the genus *Nitrospira*. Enriched and isolated from a freshwater pond sediment, NS4 culture exhibits remarkable physiological and genomic traits that shed light on NOB survival strategies in low pH conditions. NS4 culture demonstrates the optimal growth at pH 6 and 0.5 mM nitrite concentration, with a maximum growth rate of 0.62 day⁻¹. Kinetic analyses reveal a high affinity for nitrite (*K_m_*_(app)_ = 4.02 µM), suggesting adaptation to oligotrophic environments. Phylogenomic and genomic-relatedness analyses position NS4 culture as a novel member within the genus *Nitrospira*, for which we propose as “*Candidatus* Nitrospira acidotolerans.” Genomic investigations indicate the presence of a complete reductive tricarboxylic acid cycle and genes for nitrite oxidation, confirming its chemolithoautotrophic lifestyle. Intriguingly, NS4 genome lacks complete pathways for cobalamin biosynthesis, implying a potential dependence on symbiotic partners for this essential cofactor. The NS4 genome harbors genes associated with acid resistance, including chaperones, transporters, and amino acid metabolism, suggesting a genetic potential for adaptation or resistance to low pH conditions. This discovery expands our understanding of NOB diversity and adaptability, offering insights into nitrogen cycling in acid-impacted ecosystems. The physiological and genomic traits of this acid-tolerant NOB open new insights for exploring the ecological significance of NOB in previously overlooked acidic habitats.

**IMPORTANCE:**

Nitrite-oxidizing bacteria (NOB) are integral to the global nitrogen cycle, yet their adaptations to acidic environments remain poorly understood. This study introduces *Candidatus* Nitrospira acidotolerans, an acid-tolerant NOB highly enriched from freshwater pond sediment. By combining physiological and genomic analyses, this work reveals unique adaptations that enable survival and nitrite oxidation under low pH conditions. Notably, the NS4 culture demonstrates high nitrite affinity and resistance to acidic stress, suggesting its ecological significance in acid-impacted ecosystems. Additionally, NS4 genomic traits reveal genetic potential of metabolic dependencies, including reliance on symbiotic partners for cobalamin synthesis. These findings expand our understanding of NOB diversity and their role in nitrogen cycling under extreme conditions, offering novel insights into microbial ecology and potential applications in managing nitrogen processes in acidic environments.

## INTRODUCTION

The nitrogen cycle forms the foundation of global biogeochemical processes, initiated by nitrogen-fixing organisms and encompassing various pathways, including aerobic nitrification and ammonification (1, 2). Aerobic nitrification consists of two distinct processes: (i) the oxidation of ammonia to nitrite, carried out by ammonia-oxidizing archaea and bacteria and (ii) the oxidation of nitrite to nitrate by nitrite-oxidizing bacteria (NOB). Recently, some members of the genus *Nitrospira*, originally recognized as nitrite oxidizers, have been found to complete aerobic ammonia oxidation (i.e., comammox, where ammonia is directly converted to nitrate) (3–5). Additionally, *Nitrospira* spp. can utilize urea or rely on hydrogen oxidation metabolism for growth (6, 7). These discoveries highlight the metabolic versatility and ecological importance of the genus *Nitrospira* while revealing gaps in our understanding of nitrification across ecosystems (8).

Despite their crucial ecological roles, NOB remain relatively under-investigated compared to ammonia oxidizers due to their slow growth rates, low environmental abundances, and cultivation challenges. However, recent advances in genomic and physiological research have expanded the phylogenetic diversity of NOB to include groups such as *Candidatus* (*Ca*.) Nitrotoga (9), *Nitrolancea* (10), *Ca*. Nitromaritima (11), *Ca*. Nitrocaldera, *Ca*. Nitrotheca (12), *Ca*. Nitrohelix, *Ca*. Nitronauta (13), and *Ca*. Nitrosediminicola (14).

The genus *Nitrospira*, belonging to the phylum Nitrospirota, comprises seven phylogenetic lineages (3, 15, 16). *Nitrospira* is recognized as a dominant NOB in both natural and engineered systems, ranging from marine and terrestrial environments to wastewater treatment plants. While its ubiquitous distribution across diverse environments is well-documented, our understanding of NOB under acidic conditions remains limited (17, 18). In particular, little is known about the presence and survival strategies of NOB in extreme pH environments (i.e., acidic or alkaline), likely due to the challenges posed by the availability of ammonia and nitrite as energy sources under such conditions (3, 19). Nevertheless, recent studies have described a few nitrifiers, especially NOB, from low- or high-pH ecosystems (20–23) shedding light on elusive nitrification processes in extreme pH environments. While studies on ammonia oxidizers in low-pH environments have gradually increased, they remain fewer than those under high-pH conditions (24–27). Moreover, research on NOB in acidic environments is still extremely limited and underexplored.

We hypothesize the presence of acidophilic or acid-tolerant NOB and aim to decipher their physiological and genomic traits. Cultivating NOB under acidic conditions presents distinct challenges, including potential substrate limitations caused by free nitrous acid formation at low pH (pKa = 3.3 for nitrite) (17). Understanding how NOB adapt to or resist acidic environments has important implications for managing nitrogen cycling in unique habitats such as acid mine drainage.

Collectively, this study seeks to address significant knowledge gaps by cultivating NOB under acidic culture conditions and comprehensively analyzing their physiological characteristics, substrate kinetics, and genomic traits. Through these combined approaches, we aim to elucidate the ecological strategies that enable NOB to thrive in acid-impacted ecosystems.

## MATERIALS AND METHODS

### Enrichment and isolation of nitrite-oxidizing bacterium

The pond, which is named as Namsaengi pond, Jeju Island, sediment samples were collected using a box core sampler. The sampling depth was 0.5 m of the bottom of water (2 m; 33.32′00″ N 126.36′51″ E). Before inoculation, most debris such as burial plants or stones were eliminated. The sediment sample (pH 5.5–6.0) was inoculated into artificial freshwater (AFW) medium and incubated at 26°C under dark and static culture conditions. For nitrite-oxidizing enrichment, it was initially started with 0.1 mM nitrite as the sole energy and nitrogen sources, 1 mM sodium bicarbonate as a carbon source, 0.1 mM potassium phosphate, 1 × trace element mixture (composition per liter: 100 mg MnCl_2_·4H_2_O, 30 mg H_3_BO_3_, 36 mg Na_2_MoO_4_·2H_2_O, 2 mg CuCl_2_·2H_2_O, 24 mg NiCl_2_·6H_2_O, 190 mg CoCl_2_·6H_2_O, 144 g ZnSO_4_·7H_2_O)(28), and 1 × vitamin solution (28). The components of the AFW medium were described by Kim et al. (29). The pH of the medium was adjusted to 4.0 and 5.0 in the presence of the following buffers: 5 mM HOMO-PIPES (pH 3.2) and MES (pH 6.0), respectively, with NaOH or HCl (30). After the initial enrichment for 1 month of enrichment, during which nitrite oxidation activity was observed, 5% (vol/vol) of the total culture was routinely transferred to fresh medium under the same enrichment culture conditions every 2 weeks. To obtain clonal cultivation and/or isolation, the enriched NOB culture was serially diluted to 10^−7^ at least 3 times. Unless otherwise stated, the enriched culture was routinely subcultured by transferring 5% (vol/vol) into sterile AFW medium buffered with MES and supplemented with 0.5 mM nitrite (adjusted with pH 6.0) as the sole energy source. The pH of the culture medium can be adjusted using either 1N hydrochloric acid or 1N potassium hydroxide. Nitrite consumption was determined and monitored by Griess color reagent (31). Nitrite was nearly stoichiometrically converted to nitrate during the cultivation period, and nitrate concentrations were determined using the vanadium (III) chloride reduction method (32). During enrichment cultivation, we performed PCR screening for the alpha (*nxrA*) and beta (*nxrB*) subunits of nitrite oxidoreductase using genomic DNA (gDNA) extracted from the cultures. The primer sets used were for *nxrA*, F1norA (5′-CAGACCGACGTGTGCGAAAG-3′) and R1norA (5′-TCYACAAGGAACGGAAGGTC-3′); and for *nxrB*, nxrB169f (5′-TACATGTGGTGGAACA-3′) and nxrB638r (5′-CGGTTCTGGTCRATCA-3′)(33, 34). Finally, the *nxrB* gene, which belongs to the genus *Nitrospira*, was amplified in the culture tubes enriched in this study. In parallel, to determine whether the enriched culture represented a single *Nitrospira* lineage (i.e., clonal cultivation), an *nxrB* gene clone library was generated, and 10 clones were sequenced using the specific primer set (nxrB169f and nxrB638r) (33, 35).

### Growth experiments and abundance estimation

To examine the effect of organic substrates on the nitrite-oxidation activity of the enriched culture, the medium was supplemented with peptone, yeast extract, and casamino acids, each at a final concentration of 0.1% (wt/vol). To determine the optimal pH (5 to 9) and nitrite concentration (0 to 0.7 mM) for the enrichment culture, nitrite oxidation rates were estimated in quadruplicate under the same culture conditions described above, except for the varied pH or nitrite levels. For each experimental condition, cultures were sampled daily to monitor nitrite consumption. The nitrite oxidation rate was calculated based on the period during which complete nitrite depletion was observed. At the endpoint of nitrite depletion, culture supernatants and pellets were collected and used for nitrite quantification and quantitative PCR (qPCR) targeting the 16S rRNA gene, as described below.

qPCR was performed as described previously (31). Briefly, the 16S rRNA gene copy number per milliliter of culture sample was estimated using the CFX Connect real-time system (Bio-Rad Laboratories, Hercules, CA) and built-in CFX manager software (v 3.0). For amplification, *Nitrospira*-specific primer set was used: Ntspa91F_up (5′-TAMRGVGGCRMACGGGTG-3′) and Ntspa662R_up (5′-CGCTACACCGGGAATTCC-3′). In addition, to estimate the abundance of total bacterial cells, the bacterial 16S rRNA gene was amplified by primers bac518F (5′-CCAGCAGCCGCGGTAAT-3′) and bac786R (5’- CTACCAGGGTATCTAATC-3′), following our previous study (31). The used primer set was slightly modified in this study to increase coverage of the most members of the genus *Nitrospira*. After qPCR, the specificity of the amplified fragments was verified by analyzing melting curves and checking the size of reaction product via agarose gel electrophoresis. All measurements for 16S rRNA gene copy number were determined in triplicate for each culture sample.

### Kinetics of nitrite oxidation

The kinetic constants of the culture were determined from oxygen consumption measurements using a fiber-optical oxygen meter (FireString-O2, PyroScience GmbH, Germany). Data were obtained using the built-in software PyroWorkbench (PyroScience GmbH, Germany). To concentrate biomass for kinetic property analysis, actively growing cells (approximately early-stationary-phase, determined by nitrite consumption) were filtered from 500 mL of culture and re-harvested. Prior to kinetic investigation, the concentrated cells were carefully washed and resuspended in fresh AFW medium without nitrite. The resuspended biomass was transferred to a 4 mL glass oxygen-respiration vial containing a stir bar. All experiments were conducted at 26°C with 150 rpm stirring. The oxygen sensor was allowed to equilibrate for 30 min to ensure signal stability. Subsequently, nitrite from stock solution was carefully injected using a syringe. Measurements were performed on at least three independently grown cultures. Nitrite consumption was calculated from oxygen consumption using a 1:0.5 ratio of nitrite oxidation to oxygen consumption. Oxygen uptake rates were plotted against total nitrite concentration using GraphPad Prism 10 (GraphPad Software, Boston, MA). Kinetic characteristics were obtained by fitting the data to a Michaelis-Menten kinetic model using the equation: *V* = (*V*max · [*S*])/(*K*m + [*S*]). Here, *V* represents the oxygen-consumption activity, *V*max is the maximum specific activity (μmol/h), *K*m is the half-saturation constant for nitrite oxidation (μM), and [*S*] denotes the nitrite concentration (μM). Values of generation time and growth rate (i.e., nitrite oxidation rate) were calculated from the exponential growth phase as described previously (31, 35).

### Genome sequencing and annotation of enriched nitrite-oxidizing bacterium

For metagenome sequencing of the enrichment culture, cells were harvested by filtration (Super Membrane disc filters, 0.22 micron pore-size; Pall Life Sciences, Ann Arbor, MI). Genomic DNA (gDNA) was isolated from the filters from completely oxidized nitrite culture (300 mL volume) through bead-beating commercial gDNA extraction kit (DNeasy PowerMax Soil Kit, Qiagen, Mo Bio Laboratories, Hilden, Germany) according to the manufacturer’s instructions. The quality and quantity of the extracted gDNA was estimated by agarose gel (0.8%, wt/vol) electrophoresis with SYBR staining and DS-11 Plus Spectrophotometer (DeNovix, Inc., Wilmington, DE). For sequencing, a DNA library was prepared following the protocol provided by sequencing company (Novogene) for the sample sequenced by NovaSeq PE 150 settled at Novogene Inc. (Singapore).

### Genome assembly, binning, annotation, and analysis

About 40.2 Gbp of raw reads were obtained from the sample. The raw reads were *de novo* assembled, and binning was conducted according to our previous studies (29, 31) using SPAdes (v3.15.5) and MetaBAT2. 4336 contigs of >1 kbp were used for binning and a total of 9 bins were obtained after low-quality and completeness elimination. The completeness and contamination for all MAGs were estimated by CheckM2 (v1.0.2) (36). Genes for each contig of the metagenome-assembled genomes (MAGs) selected as a high quality were predicted using prokka (v1.14.5) and taxonomy for all MAGs were assigned based on the Genome Taxonomy DataBase (GTDB, version r214) with default parameters. Finally, based on the resultant in binning and annotation, MAG related to the genus lineage II “Nitrospria_D*”* was clearly identified using GTDB-Tk and denominated as MAG NS4 (or NS4 genome). Along with MAG NS4, heterotrophic bacterial MAGs (*n* = 8) affiliated with the phyla Pseudomonadota, Actinomycetota, and Bacteroidota were also recovered through GTDB analysis despite a thorough reduction (or elimination) of heterotrophic bacteria using the dilution-to-extinction method.

For phylogenomic tree construction and comparative genomic analysis, we retrieved 43 additional publicly available genomes or MAGs assigned to the genus “Nitrospira_D” [e.g., UBA6439 (GCA_002435325), ST-bin5 (GCA_002083555), and *Nitrospira lenta* BS10 (GCF_900403705)] from the GTDB (accessed in July 2024). In addition to these, MAGs from our previous study (31) were also included in the analysis. In addition, the genes of the compared genomes were annotated by prokka and prodigal, and its functions were assigned BlastKOALA for parallel analyses. A total of 49 genomes including 5 genomes belonging to the phylum Aquificota as an outgroup were analyzed with single-copy marker genes (*n* = 194) as previously described (31). Finally, 48 genomes containing at least 50% of markers (*n* = 97) were selected. A maximum likelihood tree was generated through IQ-TREE (v2.2.7) with the best-fitting substitution model (GTR + F + I + R5 chosen automatically by ModelFinder in this study) assessed with ultrafast bootstrapping (-ufboot 1000) (29, 31). Average nucleotide identify (ANI) and average amino acid identity (AAI) between all MAGs selected in this study were determined as our previously described (29, 31). The taxonomy for the MAG NS4 was defined based on the standards (31, 37). For a comparative genome analysis, the GFF3 files were generated using prokka (see above) with default parameters and analyzed with Roary (v3.13) (38). In parallel, anvi’o (v8) was also used to perform pangenome profiling (39). To estimate the recombination and horizontal gene transfer (HGT) events, ClonalFrameML and MetaCHIP were used, respectively (40, 41).

### Evaluation of metabolic and physiological potential

To assign and/or predict the function and classification for the predicted coding sequences (CDSs), the CDSs were compared against the KEGG Orthology (KO numbers) and Clusters of Orthologous Groups (COGs) data sets via BlastKOALA and RPS-BLAST (reverse-position-specific BLAST), respectively (42, 43). Additionally, if necessary, metabolic modules and pathways based on KEGG or functional prediction were used by EggNOG-mapper v2 (2.1.12) (44), Kofam (45), and Pfam (46). For identification of CRISRP arrays and Cas proteins, the webserver-based CRISPRCasFinder (https://crisprcas.i2bc.paris-saclay.fr/) was used with default parameters (47).

## RESULTS AND DISCUSSION

### Enrichment of the acid-tolerant nitrite oxidizer

Several enrichment cultures having nitrite oxidation activity were obtained from freshwater sediment from pond on Jeju Island, Republic of Korea. To enhance the activity for the chemolithoautotrophic nitrite-oxidizer against other heterotrophic microorganisms, the cultures were only supplied with nitrite as the sole energy source and bicarbonate as the sole carbon source in each culture tube. The first nitrite consumption (i.e., converted to nitrate) was shown after about 2 months. We initially attempted to enrich nitrite-oxidizing microorganisms at pH 4.0. Although nitrite consumption was observed in several culture tubes, successive transfers showed declining activity. Control experiments without inoculation also exhibited nitrite depletion, suggesting rapid auto-oxidation (abiotic oxidation) of nitrite to nitrate under strongly acidic conditions (i.e., pH 4.0, data not shown) (17). In contrast, no evidence of nitrite auto-oxidation was observed at pH 5.0. We, therefore, re-enriched nitrite oxidizers at pH 5.0. Enriched cultures exhibiting nitrite oxidation activity were successively transferred to fresh medium (5%, vol/vol inoculum) every 2 weeks after nitrite was nearly stoichiometrically converted to nitrate. Stable nitrite oxidation activity was observed in a highly enriched culture, designated as NS4 (hereafter referred to as the NS4 culture). During enrichment cultivation, to obtain clonal NOB and reduce contaminated heterotrophs, the NS4 culture has been serially diluted to extinction with the activity for nitrite oxidation at least five times. We finally examined whether the culture contains the single NOB but not the others by constructing and analyzing clone library based on PCR amplified beta subunit of nitrite oxidoreductase gene (*nxrB*, 457 nt analyzed in this study)(33) (also see the Materials and Methods). The analysis revealed that there was only one unique sequence related to the environmental clone E12_P12 belonging to the genus *Nitrospira* (100% identification calculated by BLASTX) isolated from UWM biofilter sand (48).

In addition, we confirmed that the 16S rRNA gene sequence derived from MAG NS4, designed as NS4 genome, was closely related to *Nitrospira lenta* BS10 (99% similarity calculated by BLASTN), a sublineage II *Nitrospria* isolated from a municipal wastewater treatment plant (49)(Fig. S1). As mentioned above, although heterogeneous microorganisms (i.e., heterotrophs) were still present in the enriched culture, the *nxrB* clone library analysis suggests that only a single strain affiliated with the genus *Nitrospira* was enriched. Consistently, MAG NS4 was classified as a member of the genus lineage II (*Nitrospira*_*D* based on GTDB taxonomy) within the genus *Nitrospira*.

**Fig 1:**
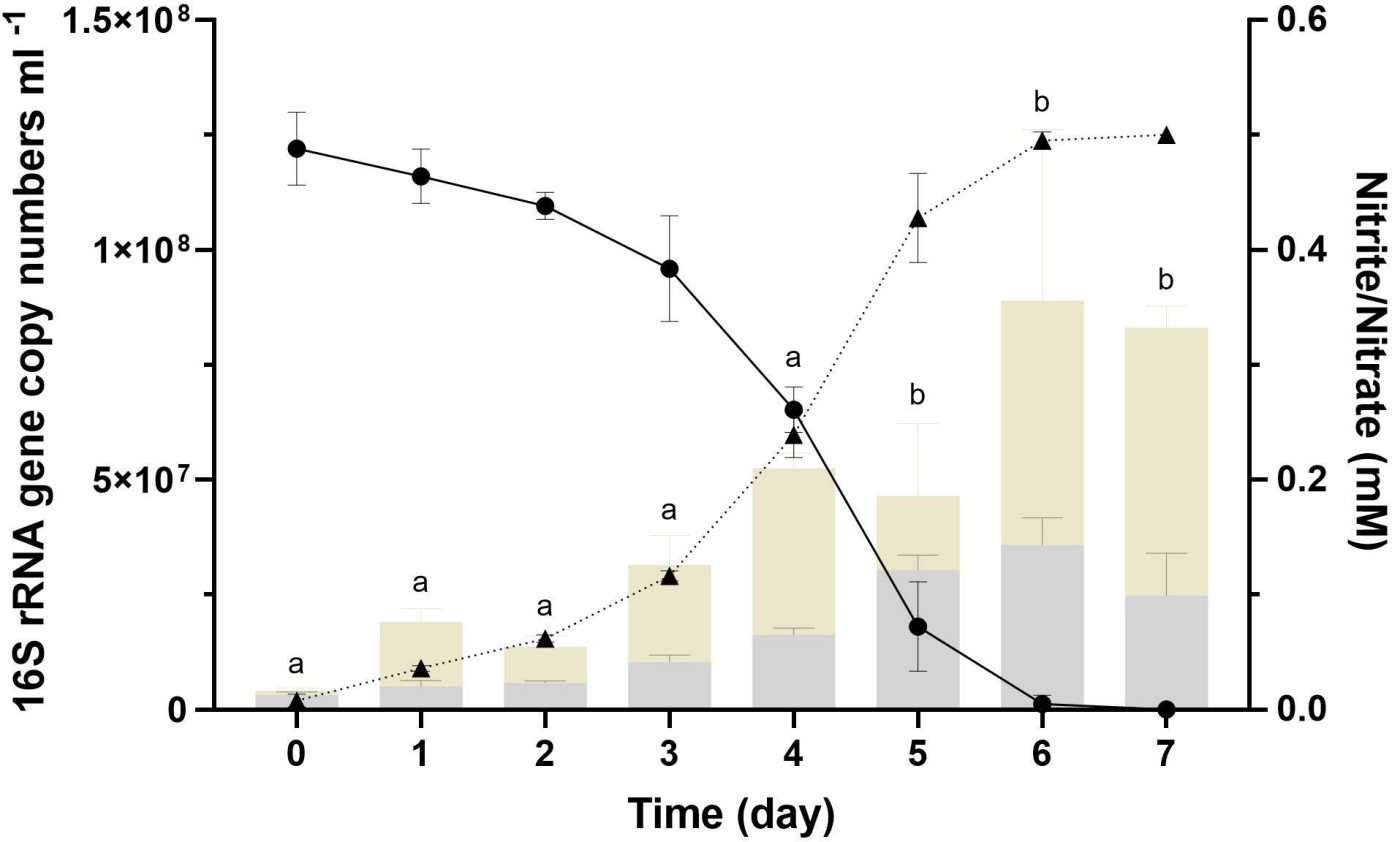
The stoichiometric relationship of nitrite consumption and growth of the NS4 culture is estimated by qPCR under the optimal culture conditions (26°C and pH 6). The culture medium was adjusted by MES buffer with an initial nitrite concentration of 0.5 mM and inoculated with 5% (vol/vol) early stationary culture and incubated under dark and static conditions. Values are shown as mean ± SD (*n* = 3). Quantification of NOB abundance based on 16S rRNA gene copy number using qPCR across sampling days [Day0 (D0)–Day7 (D7)]. Statistically significant differences (*P* < 0.05) were determined by one-way ANOVA followed by Tukey’s HSD *post hoc* test. Different letters above the bars indicate significant differences between sampling days. Light gray and light beige bars represent 16S rRNA gene copy numbers of NOB and total bacteria, respectively, in the NS4 culture.

The growth for the NS4 culture estimated by NOB-specific 16S rRNA gene copy number was observed during nitrite consumption (Fig. 1). qPCR analysis targeting the 16S rRNA gene revealed that *Nitrospira* comprised up to 65.3% of the total microbial abundance during the cultivation period, indicating its predominance in the enrichment culture. The supplied nitrite was almost stoichiometrically converted to nitrate within 1 week. While heterotrophic microorganisms in the culture showed growth when organic substrates like peptone, yeast extract, or casamino acids (0.1%, wt/vol) were present, no nitrite oxidation activity or NOB growth was detected under these conditions (data not shown). While the 16S rRNA gene sequence of NS4 was closely related to *Nitrospira lenta*, no nitrite oxidation activity was observed in the presence of organic substrates. These results suggest that NS4 likely depends on strict chemolithoautotrophy, distinguishing it physiologically from mixotrophic nitrite oxidizers [e.g., *Nitrobacter agilis* (50), *Nitrospira marina* (51)] and from *N. lenta*, which has only been suggested as mixotrophic based on genome analysis (52).

### Kinetics of nitrite oxidation

**Fig 2:**
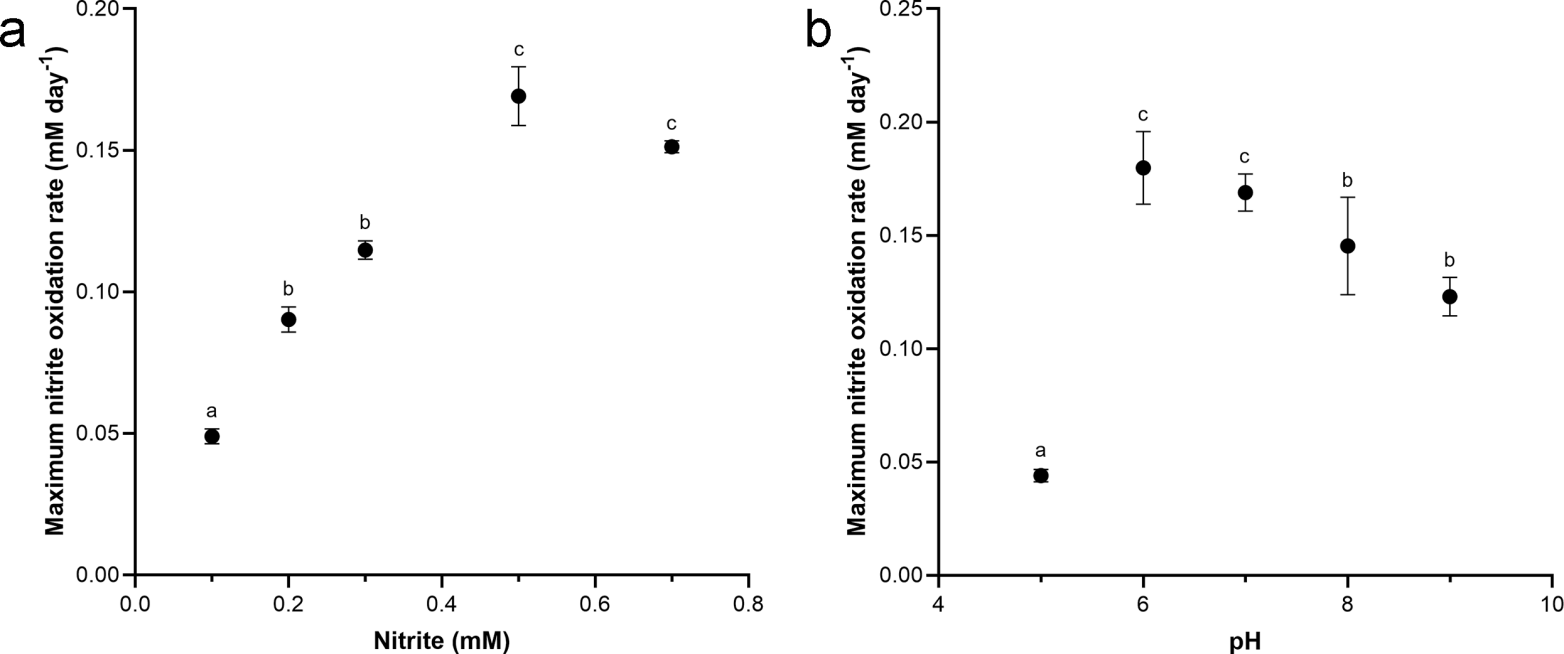
Effects of nitrite concentrations (**a**) and pH (**b**) on the nitrite oxidation activity of NS4 culture. In both plots (**a**) and (**b**), the activity of nitrite oxidation was determined by nitrite consumption. Nitrite oxidation rates were determined from time-points during nitrite consumption. Error bars indicate mean ± SD (*n* = 3). Statistically significant differences (*P* < 0.05) were determined by one-way ANOVA followed by Tukey’s HSD *post hoc* test. Different letters above the bars indicate significant differences among conditions.

To evaluate nitrite oxidation rates, NS4 culture was cultivated under various nitrite concentrations (0.1, 0.2, 0.3, 0.5, 0.7, and 1 mM) and pH levels (ranged from 5 to 10 at intervals of 1 pH unit) (Fig. 2). NS4 culture was able to oxidize nitrite in a dose-dependent manner up to 0.5 mM; however, a decrease in nitrite oxidation activity was observed (Fig. 2a), and NOB cell growth was inhibited at 0.7 mM nitrite compared to 0.5 mM, as determined by qPCR (data not shown). Nitrite oxidation was observed across a range of pH values (pH 6 to 9), with maximum activity occurring at pH 6 (Fig. 2b). However, growth inhibition (estimated by nitrite consumption) was relatively started and observed at pH 7 (Fig. 2b). The NS4 culture exhibited a slightly lower pH range compared to other characterized *Nitrospira* isolates, including *N. moscoviensis* (pH 7.6–8.0), *N. lenta* (pH 7.4–8.0), *N. marina* (pH 7.6–8.0), and *N. alkalitolerans* (pH 8.9–10.3), indicating its adaptation to mildly acidic environments (see Table S1). The optimal nitrite concentration and pH were determined to be 0.5 mM (170 µM day^−1^) and 6 (179 µM day^−1^), respectively (as determined by maximum nitrite oxidation rate). No growth of NS4 culture was observed at 1 mM nitrite and pH 10 (data not shown). Based on these observations, the growth kinetics of NS4 culture were evaluated under the optimum culture conditions (0.5 mM of nitrite and pH 6) (Fig. 1). Maximum growth rate (*µ*_max_) of NS4 culture was determined by 0.62 day^−1^ under the optimal condition. Except for *Nitrospira japonica* (0.72), the growth rate of NS4 culture was slightly higher than that of other members of the *Nitrospira* [ranged from 0.12 to 0.48 day^−1^ recalculated from Martinez-Rabert et al. (53)]. In addition, the *µ*_max_ value of NS4 culture was slightly less than that of “*Candidatus* Nitrobacter laanbroekii” NHb1 (ranged 0.70–1.17 depending on pH) recently proposed and cultivated as an acidotolerant soil nitrite oxidizer (54). This observation deduces one possibility that in acidic environments, members of both genera *Nitrospira* and *Nitrobacter* may occupy similar ecological niches, potentially leading to direct competition over nitrite oxidation. This hypothesis is supported by the comparable growth rates and pH adaptations observed in NS4 culture (*Nitrospira*) and “*Candidatus* Nitrobacter laanbroekii” NHb1, both of which demonstrate optimal growth under acidic conditions and at similar nitrite concentrations (pH 6 and 0.5 mM of nitrite). Those potential competitive relationships between NOB in low pH environments may expand more significant understanding and ecological dynamics for nitrogen cycling in acidic soils and other similar ecosystems.

Simultaneously, the nitrite oxidation rate was estimated from the batch growth experiment of NS4 culture. The specific oxidation rate of nitrite and generation time were determined by 13.43 fmol cell^−1^ day^−1^ and 1.11 day^−1^, respectively. These values were extremely less than other cultivated NOB including members of the genus *Nitrospira* (55–58). It suggests that a unique metabolic strategy that may be advantageous in resource-limited or extreme environments (i.e., low pH). These characteristics indicate that NS4 culture might occupy a specialized ecological niche (K-strategy) (59), similar to other members of the genus *Nitrospira* (58, 60), potentially contributing to nitrogen cycling in conditions where other NOB struggle to thrive. Further investigation into the genetic basis of these traits and the ecological distribution of NS4 culture could provide valuable insights into the diversity and adaptability of nitrite-oxidizing bacteria, potentially reshaping our understanding of their roles in various ecosystems.

**Fig 3:**
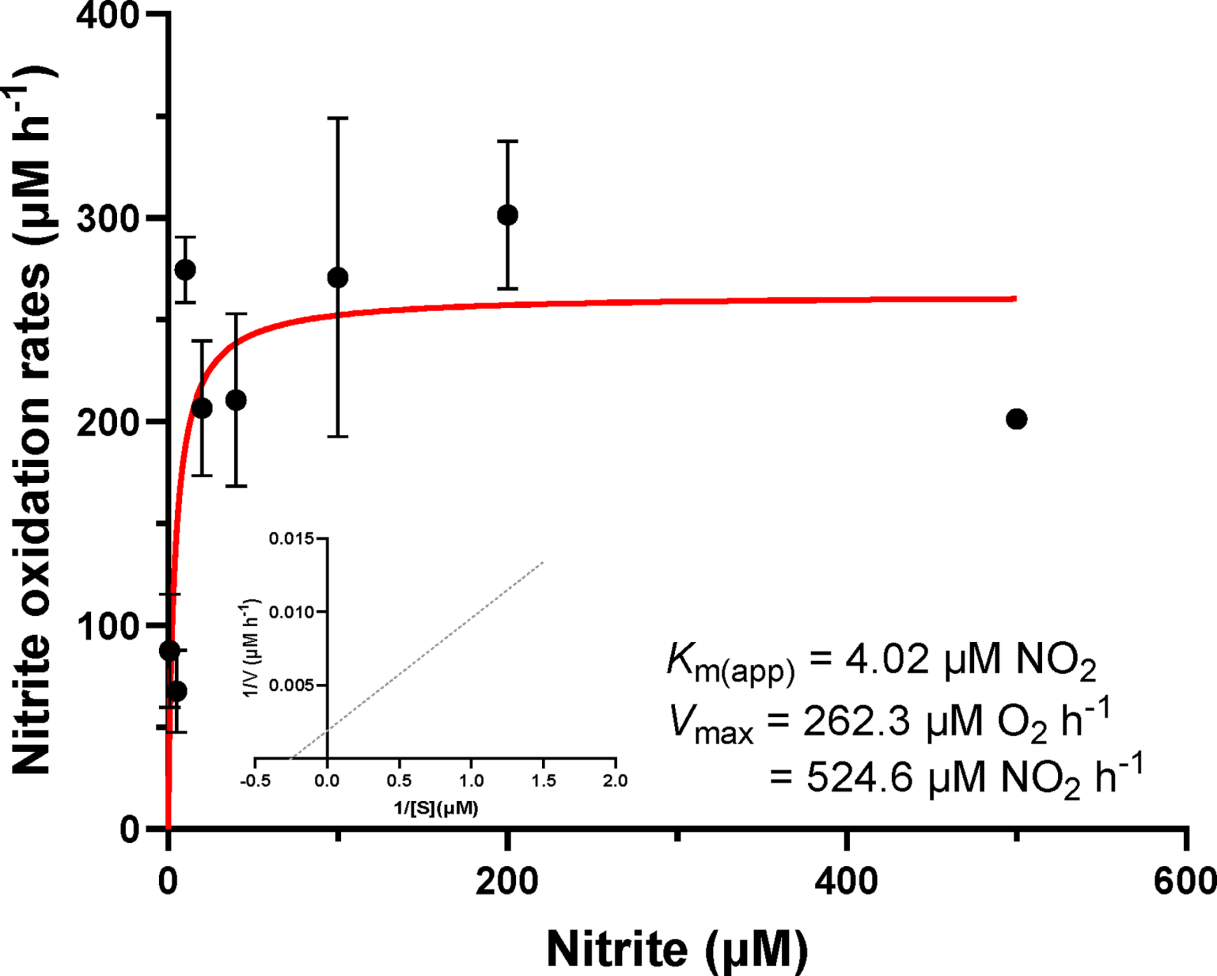
Nitrite oxidation kinetics of “Ca. Nitrospira acidotolerans” NS4 culture. Michaelis-Menten plot of oxygen uptake according to the various nitrite concentrations (µM). The experiments were performed with early-stationary-phase cells at 26°C. Data represent mean ± SD (*n* = 3). The kinetic parameters were calculated by ﬁtting a Michaelis-Menten equation to the data. Inner graph plotted by Lineweaver-Burk equation.

To further understand the unique ecological adaptation and metabolic characteristics of NS4 culture, we investigated its substrate (i.e., nitrite) affinity (Fig. 3) and oxidation kinetics (Fig. S2). We determined the Michaelis-Menten kinetics for both nitrite and oxygen to gain insights into the nitrite consumption of NS4 culture under various nitrite concentrations. Two key parameters were measured: the half-saturation constant (*K_m_*_*(*app)_) and the mean maximum oxidation rate (*V*_max_) for nitrite. The *K_m_*_(app)_ was 4.02 µM NO_2_^−^, and the *V*_max_ was 524.6 µM NO_2_^−^ h^−1^. The *K_m_*_(app)_ value of NS4 culture was compared with other *Nitrospira* species: 9 µM for “*Ca*. Nitrospira defluvii,” 9 µM for *Nitrospira moscoviensis*, 27 µM for *Nitrospira lenta* BS10, 0.81 µM of *Nitrospira* sp. KM1, 10 µM *Nitrospira japonica* NJ1, 77 µM *Nitrospira tepida* (16, 56, 58). These comparisons suggest that NS4 culture may have a competitive advantage in nitrite uptake over other *Nitrospira* species, particularly in acidic or oligotrophic environments where nitrite is present at nanomolar levels (61), due to its relatively high affinity for nitrite. In addition, the *K_m_*_(app)_ value of NS4 aligns with nitrite concentrations reported under acidic conditions (pH ≤ 5.5), where ammonia oxidation generates low but sustained nitrite levels (e.g., approximately 200 µM in soil) (62–64). These findings suggest that the NS4 culture is physiologically adapted to acidic niches characterized by limited nitrite availability.

In fact, this hypothesis is supported by substrate affinities of ammonia oxidizers, which provide nitrite to NOB in acidic environments (65–67). With the exception of Except of “*Ca*. Nitrosoglobus terrae” strain TAO100 (58.5 mM at pH 6), most ammonia oxidizers including Comammox exhibit a high affinity for ammonia ranging from 40 nM to 11 µM (65, 66), strongly suggesting the metabolic synergy of acid-tolerant ammonia oxidizers and nitrite-oxidizers including our strain.

Recent studies have highlighted the dominance of NOB under oxygen-limited conditions, emphasizing their significant role in nitrogen cycling (68, 69). Among these NOB, *Nitrospina* is predicted to occupy unique ecological niches in marine oxygen minimum zones (OMZs), where it adapts exceptionally well by oxidizing nitrite under low oxygen concentrations. For *Nitrospira*, the oxygen affinity was measured at approximately 16.9 ± 4.4 µM, while other NOB members exhibited *K_m_*_(app)_ values for oxygen ranging from 1.5 to 165.8 µM (references in 70). In contrast, the NS4 culture showed a higher oxygen *K_m_*_(app)_ value of 46.4 µM (Fig. S2), which was comparable to those reported for the members of *Nitrobacter* (ranging from 10.9 to 165.8) (53, 70). These findings suggest that, although NOB are aerobic nitrite oxidizers, they are adapted to lower oxygen concentrations, underscoring their crucial contribution to nitrogen cycling in oxygen-limited environments (71). Given the unique metabolic characteristics of NS4 culture revealed in this study, further in-depth genomic investigations are warranted to unravel the genetic potential underlying its remarkable substrate affinity and environmental adaptability.

### General genomic description and phylogenomic analysis

Assembly and binning of metagenomic reads of the enrichment culture showed that one high-quality MAG (named as MAG NS4 or NS4 genome) comprised of 15 contigs, corresponding to the putative NOB (i.e., *Nitrospira*) genome (98.97% completeness and 1.37% contamination estimated by CheckM2). To verify if the MAG NS4 belongs to the genus *Nitrospira*, a phylogenomics tree calculated with maximum likelihood was constructed with publicly genome sequences that fell within the genus *Nitrospira* selected in this study. Aside from MAG NS4, eight additional MAGs were recovered and classified into five *Pseudomonadota*, two *Actinomycetota*, and one *Bacteroidota* (see Supporting Data).

**Fig 4:**
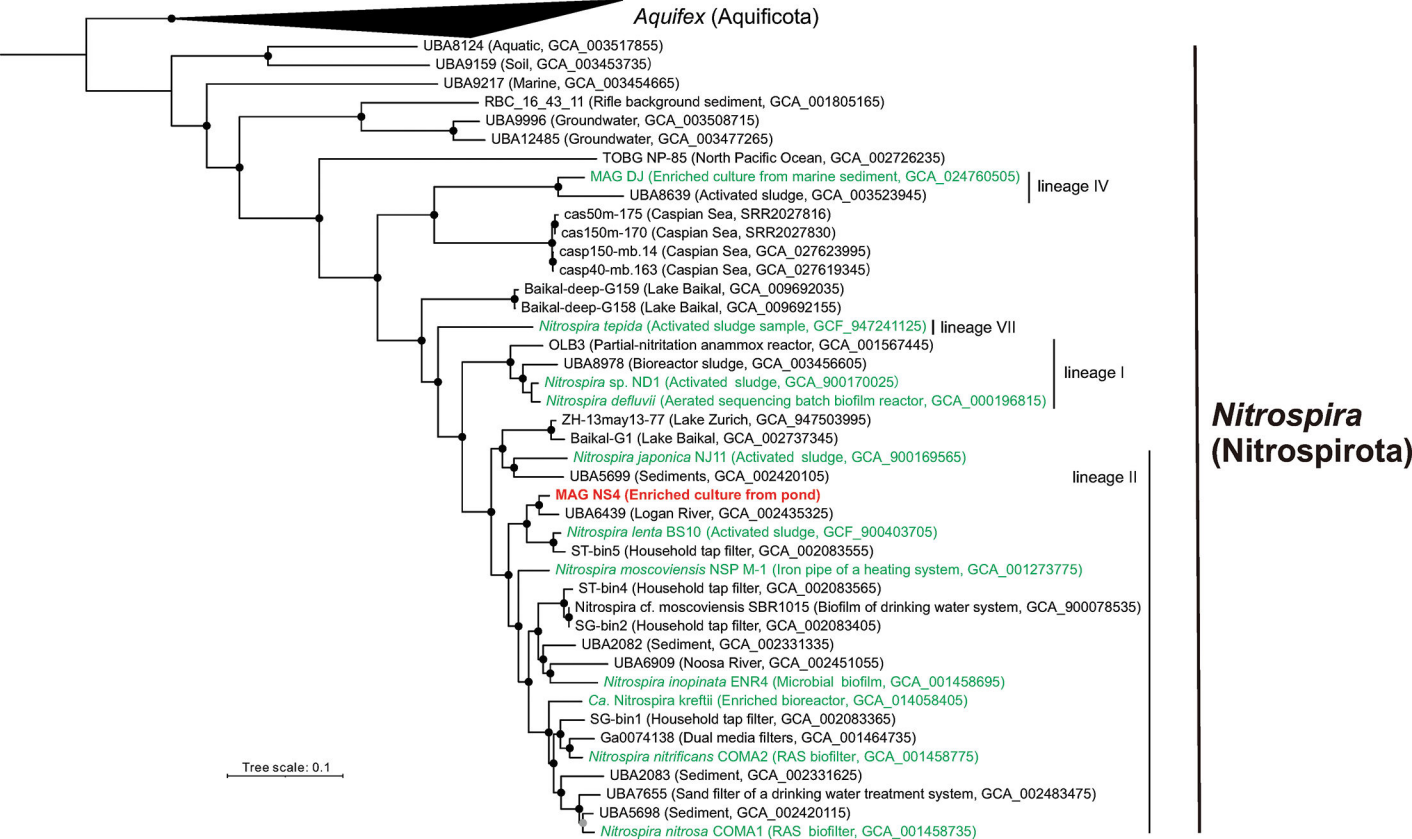
Phylogenomic tree of the members of the genus *Nitrospira*. Whole-genome phylogenies based on a maximum likelihood (phylogenomic) tree inferred. The NS4 genome and cultured *Nitrospira* spp. are highlighted in red boldface and green, respectively. The strength of support for internal nodes was assessed by performing bootstrap replicates, with the obtained values shown as closed circles. A phylogenomic tree was generated using iqtree2 with 1,000 ultrafast bootstraps and the best-fit model of nucleotide substitution (GTR+F+I+R5) chosen according to Bayesian Information Criterion. A midpoint root was used when selecting outgroup sequences belonging to the genus *Aquifex*.

Based on the similarity to the 16S rRNA gene sequence derived from MAG-based and genome sequencing, NS4 genome was closely related to the *Nitrospira lenta* BS10 (Fig. 4 and Fig. S1). Nevertheless, NS4 genome was identified as a novel species due to the values for ANI (82.02% and 75.92%) and AAI (83.41% and 76.60%) to UBA6493 (GCA_002435325) harvested from Logan River classified to the genus *Nitrospira* and *N. lenta* strain BS10, respectively, supporting the cut-off of critical value (i.e., 95%) for species delimitation (37, 72, 73). Therefore, we designated to this NS4 culture as “*Ca*. Nitrospira acidotolerans,” representing aerobic nitrite-oxidizer in acidic environment.

The estimated genome size of NS4 genome was 3.67 Mbp with 59.69% GC content. From a total of 3,606 predicted genes by prokka (see also M&M), the MAG NS4 contains 3,555 CDSs and 50 RNAs (47 tRNAs and 3 rRNAs) with 87.78% coding density (see the additional data). A total of 2,253 and 2,029 CDSs from MAG NS4 were categorized in the COG and KEGG databases, respectively. The dominant categories of COG were identified with Cell wall/membrane/envelope biogenesis (M, 9.63%), followed by translation, ribosomal structure and biogenesis (J, 8.88%), energy production and conversion (C, 8.03%), signal transduction mechanisms (T, 7.32%), posttranslational modification, protein turnover, chaperones (O, 6.44%), and amino acid transport and metabolism (E, 5.99%). General function prediction only (R) and function unknown (S) were calculated with 8.61% and 3.99%, respectively (Fig. S3). Excluding the unassigned genes (approximately 36%), a notable fraction of the annotated genes belonged to categories related to energy production and cellular responses (e.g., COG categories C, T, and O), suggesting potential physiological adaptations (e.g., to acidic conditions). In addition, a small number of genes associated with defense mechanisms (category V, 2.89%) and mobilome elements such as prophages and transposons (category X, 0.80%) were also identified in the MAG NS4. Similar to the genomes of *N. japonica* NJ1, *N. defluvii,* and *N. lenta* BS10 (52, 71, 74), only one CRISPR array was predicted in the genome of NS4. In contrast, other *Nitrospira* species (i.e., *Nitrospira* sp. ND1 and *N. tepida*) possessed multiple CRISPR-Cas systems (16, 74).

Although this analysis was based on an incomplete genome, MAG NS4 exhibited high completeness as previously mentioned. Nevertheless, we cautiously speculated that phage defense strategies might differ among members of the genus *Nitrospira* and that certain species may experience varying levels of ecological stress from predators or infections (e.g., phages) depending on their environmental habitats. Furthermore, some *Nitrospira* species, including NS4, appeared to have limited potential for recombination derived from horizontal gene transfer (75). These findings may encourage future exploration into the interactions between microbes and phages.

### Central carbon metabolism and energetics

The genome of NS4 culture encodes genes (*nxr*) for nitrite oxidation and contains a complete set of genes for the reductive tricarboxylic acid (rTCA) cycle, consistent with other *Nitrospira* genomes. Specifically, genes for citrate synthase, isocitrate dehydrogenase, malate dehydrogenase, ATP-dependent citrate lyase, and pyruvate:ferredoxin oxidoreductase (Por), key enzymes in both oxidative and reductive TCA cycles, were identified in the NS4 genome. Although the automated annotation did not detect a gene encoding 2-oxoglutarate:ferredoxin oxidoreductase (OGOR) which firstly reported in a *Nitrospira* genome (71), manual homology-based analysis (BLASTp) revealed a putative homolog, based on alignment with the *N. defluvii* genome. Additionally, the absence of genes involved in the Calvin-Benson-Bassham cycle, such as ribulose-1,5-bisphosphate carboxylase and ribulose-5-phosphate kinase, was confirmed. Genes (*korAB*) for the isoenzyme of OGOR were not detected in the NS4 genome, whereas *Nitrospira* clades encode kor genes (76).

Notably, for energetic features, two genes related to high-affinity oxygen utilization—cytochrome *c* oxidase *cbb*_3_-type subunit III (*ccoP*) and cytochrome *d* ubiquinol oxidase subunit I (*cydA*)—were identified in the NS4 genome (31). Taken together, these findings suggest that NS4 culture may be capable of respiration under oxygen-limited conditions similar to other nitrifiers adapted to microaerobic environments. This is supported by the presence of Por and terminal oxidases encoded in its genome (71, 77).

Based on the BLASTP analysis against GenBank of NCBI, the NxrA protein for nitrite oxidation in NS4 culture exhibits high similarity to *Nitrospira lenta* (97.66%) and *Nitrospira* sp. MAG C18_metabat.bin.90 C18_k141_445403, which was reconstructed from a groundwater metagenome (MDP3092842; 97.66%). In contrast, the NxrB protein showed 100% similarity to *Nitrospira* sp. MAG STL-NOB7 C1.118.fa-11, reconstructed from a full-scale hybrid biological wastewater treatment system metagenome (MFO0705359), and 99.77% similarity to *Nitrospira moscoviensis* (WP_053378141).

While most other members of the genus *Nitrospira* harbor multiple copies of the NXR operon, only a single copy of the two genes (*nxrA* and *nxrB*) was identified in MAG NS4. Although only single copies of *nxrA* and *nxrB* were identified in MAG NS4, it is possible that additional copies may have been merged during the assembly process due to the high sequence similarity among these genes in *Nitrospira* genomes (33, 71). Two genes were located on separate contigs. While no gene was explicitly annotated as *nxrC* in MAG NS4, we manually identified one gene encoding an ethylbenzene dehydrogenase-related protein, which may function as a NxrC-like subunit. The amino acid sequence of the putative NxrC in MAG NS4 showed 63.1% identity to *Nitrospira* sp. ND1 and *N. defluvii*, and 81.1% identity to *N. lenta*. Despite the MAG NS4 being nearly complete and of high quality, it remained difficult to confirm the presence of multiple copies of the NXR operon in the genome. However, several copies of the sigma-54 interaction domain (PF00158) were detected in its genome (56).

Additionally, genes associated with denitrification, as well as dissimilatory and assimilatory nitrate reduction (*nirCDAK*, *norBQ*, and *nrfH*), were identified in the NS4 genome. This observation aligns with previous findings that these genes are also present in the genomes of other members of the genus *Nitrospira*, including Comammox species such as *Nitrospira inopinata* (4, 7, 16, 56, 71, 74). The NS4 genome contained two nitrite reductase-related genes: *nirA* (involved in assimilation) and a *nrfH*-like gene (potential involved in dissimilation) although *nrfA* was absent. In addition to previous reports of both genes in only two *Nitrospira* genomes, *Nitrospira* sp. ND1 and *N. inopinata*, Kop et al. (78) recently demonstrated that *nrfAH* genes are more broadly distributed across multiple *Nitrospira* genomes. This suggests that these genes may be more common within the genus *Nitrospira* and are not restricted to a few strains. In particular, the *nrfH*-like gene in the NS4 genome was annotated as a cytochrome *c7*-like protein, belonging to the NapC/NirT family cytochrome *c* (or cytochrome *c3* family), based on BLASTp analysis. Homologs of this gene were also found in other *Nitrospira* genomes, including *N. lenta* (WP_121990035; 85.96% identity), *N. japonica* (WP_080885040; 77.02%), *N. moscoviensis* (WP_053380978; 76.60%), and *N. defluvii* (CBK43026; 75.11%). Interestingly, Simon et al. (79) reported that NapC/NirT-type cytochrome *c* (NrfH) functions as an electron mediator from the quinol pool to nitrite reductase. Furthermore, Campeciño et al. (80) identified a novel NrfA subclass in *Geobacter lovleyi*, highlighting the potential diversity of nitrite-reducing systems. Therefore, it may not be excluded that novel subclasses of genes involved in the reduction of nitrite to ammonium exist within the genus *Nitrospira* including NS4.

The NS4 genome contains genes (*hyfBCDF*) encoding the group 4 [NiFe]-hydrogenase, part of the so called Ancient Enzyme Complexes, which are also found in other *Nitrospira* genomes, such as *N. mosoviensis*, *N. defluvii*, and *N. japonica*. However, the large subunit of the hydrogenase in NS4 genome lacked the conserved CxxCH motif required for nickel-iron cofactor binding, suggesting that it may not function in classical hydrogen metabolism. In addition, Koch et al. (6) reported that the group 4 hydrogenase in *N. moscoviensis* is not expressed under hydrogen-oxidizing conditions, supporting the hypothesis that this complex may serve alternative roles. Although it has proposed the reversible disproportionation (formate to hydrogen and carbon dioxide) as a possible function (81), it has also been shown that oxygen-sensitive hydrogenases, including group 4 types, are inactivated under oxic conditions (82, 83). Moreover, a variety of group 4 hydrogenase subtypes have been reported (84), and certain complexes may contribute to energy conservation rather than hydrogen metabolism *per se*—particularly under oxygen-limited conditions (85–87). Additionally, single-copy genes for a membrane-bound hydrogenase (*mbhJ*) and a formate hydrogenlyase (*hycE*) were identified in MAG NS4. Collectively, these findings highlight the need for further investigation into the physiological roles of group 4 [NiFe] hydrogenases in *Nitrospira*, which may prioritize energy conversion under oxygen-limited conditions, rather than functioning in classical hydrogen-related metabolism (6, 31, 88, 89).

### Other alternative metabolisms

In general, members of the genus *Nitrospira* exhibit metabolic versatility, enabling them to utilize a wide range of substrates for energy conservation and/or assimilation. NS4 genome possesses the gene (*cynS*) for cyanate hydratase, which converts cyanate to ammonia and carbon dioxide (90). Additionally, the NS4 genome encodes an Amt-type ammonium transporter and genes (*glnKD* and *glnA*) involved in PII signal transduction proteins and glutamine synthetase, respectively (91). These results indicate that ammonia assimilation may occur in NS4, as evidenced by the presence of the *nirA* gene (56, 77). However, genes related to utilization of other forms of nitrogen (e.g., urea) were not detected in the NS4 genome. In contrast, such genes, including *ureC*, have been identified in the genome of *N. lenta*, which is closely related to NS4 and possesses a complete gene repertoire (*urtABCDE*) for a high-affinity urea transporter, as well as in other *Nitrospira* genomes reported such as *N. moscoviensis* (7, 52, 74).

Notably, genes (*prpCBD*) for the methylcitrate cycle, which regenerates pyruvate, succinate, and oxaloacetate and is closely related to the TCA and glyoxylate cycles, were found in the NS4 genome. These genes are also present in other *Nitrospira* strains and *Ca*. Nitromaritima RS (11). However, the genes were identified to be expressed at extremely low levels in *N. moscoviensis* M-1 under stable nitrite-oxidizing conditions (92), suggesting that the cycles may not be active under standard nitrifying conditions. Furthermore, the NS4 genome contains complete gene sets for glycogen biosynthesis and degradation pathways (7, 31), which are also commonly identified in most *Nitrospria* genomes (6, 31, 74, 89, 93). These findings suggest that NS4 culture has the genetic potential to utilize organic matter as alternative carbon or energy sources (references in 52) although heterotrophic growth was not observed. Moreover, transcriptomic analyses of *Nitrospira* strains have reported that these genes were either not expressed or expressed at very low levels (88, 92). Taken together, these observations highlight the need to further refine our understanding of the metabolism and physiological characteristics of the genus *Nitrospira*.

### Genomic resistance and/or adaptation to the acidic environment

The optimal pH for nitrite oxidation in NS4 culture was lower than that observed in canonical *Nitrospira* strains isolated from neutral (e.g., activated sludge) or alkaline (e.g., saline-alkaline lake) environments. This finding supports the hypothesis that NS4 has adapted to acidic niches through potentially unique genetic mechanisms. The genome of NS4 culture contains a considerable number of genes for acid resistance; for example, its CDSs classified as chaperones and amino acid transport and metabolism comprise up to 12% of its genome (94). The encoded genes include potassium transporter (*kdpABCDE*), 3-oxoacyl-[acyl-carrier-protein] synthetase (*fabBH*), argininosuccinate synthase (*argG*), argininosuccinate lyase (*argH*), methylmalonyl-CoA mutase (*mcmA1A2*), 5-methyltetrahydrofolate—homocysteine methyltransferase (*metH*), molybdopterin molybdotransferase (*moeA*), ATP-binding cassette transporters (ABCs) for iron (*AfuABC*), sulfate/thiosulfate (*cycPUWA*), and phosphate (*pstSCAB*), and chaperones (*dnaK*, *groES*, *groEL*, *dnaJ*, *grpE*, *hsp20*, and *cspA*). However, the NS4 genome lacks putative genes encoding key proteins involved in pH homeostasis under acidic conditions, including ActS/PrrB/RegB family redox-sensitive histidine kinases, the acid tolerance regulatory protein (ActR), and carbonic anhydrase (CA) (65, 95). Additionally, the NS4 genome harbored genes for two types of superoxide dismutase (Fe-Mn and Cu-Zn family), thioredoxin, excinuclease ABC (*uvrABC*), ATP-dependent DNA helicase (*uvrD*), and cell cycle sensor histidine kinase and response regulator (*cckA*). However, the NS4 genome lacks genes for low-affinity potassium transporter (*kup*) Na^+^/H^+^ antiporter (*mnh*), Ca^2+^/H^+^ antiporter (*chaA*/*CAX*), cation/H^+^ antiporter (*yrbG* or *nhaK*), K^+^/H^+^ antiporter (*cvrA*/*nhaP2*), and ABC transporters for manganese, copper, magnesium, and zinc, which are involved in maintaining ion balance and/or resisting ion flux (20, 21, 24, 31, 65, 96, 97). No genes involved in flagellum synthesis were found in the NS4 genome. Nevertheless, the NS4 genome encodes proteins for methyl-accepting receptor HlyB-like (*mcp*), chemotaxis proteins (*cheWY* and *motAB*), and twitching motility via a type IV pilus (complete repertory), unlike most nitrifying microorganisms (31). This genetic potential for motility may provide an advantage under low pH conditions, particularly in environments with ion flux transition zones.

Intriguingly, most previous genome investigations of NOB suggest that they either synthesize or take up cobalamin (vitamin B_12_), a vital cofactor for many organisms (98). Especially, many prokaryotes including NOB, there are three known cobalamin biosynthesis processes: aerobic, anaerobic, and a salvage pathway involving corrinoid uptake (31, 89, 99, 100). However, the genome of NS4 culture encodes only a few genes related to cobalamin biosynthesis, including adenosylcobyric acid synthase (CobQ/CbiP), cob(I)alamin adenosyltransferase (MMAB), precorrin-2 dehydrogenase (SirC), and uroporphyrinogen III methyltransferase/synthase (CobA-HemD). Additionally, genes for a cobalamin transporter (*btuBDCF*) were identified in the NS4 genome. This suggests that NS4 culture may require symbiotic partners, such as ammonia-oxidizing archaea, to provide cobalamin for its growth, differing from other NOB (101, 102). Simultaneously, the absence of biosynthetic pathways, such as cobalamin, suggests a potential metabolic interaction with a cobalamin producer. The type of interaction is unknown, but might be consistent with the Black Queen Hypothesis, which proposes that some microorganisms lose energetically costly biosynthetic functions and instead rely on metabolites produced by neighboring community members (103).

### Comparative genomics and genome plasticity

It is well known that members of the genus Nitrospira exhibit metabolic versatility due to their unique gene contents. The observed metabolic versatility may reflect underlying genetic plasticity resulting from HGT and recombination events. To evaluate this genetic flexibility, we selected seven genomes, MAG NS4 and six genomes from isolated canonical NOB strains (*N. defluvii*, *N. lenta*, *N. moscoviensis*, *Nitrospira* sp. ND1, *N. tepida*, and *N. alkalitolerans*), and conducted comparative genome analyses. We first performed a comparative genome analysis using Roary. Among a total of 25,826 genes, 612 genes were classified into the “shell” group (<95%) and 25,214 genes into the “cloud” group (<15%). No genes were assigned to the “core” (≥99%) or “soft core” (≥95%) categories. This indicates that the majority of genes belong to non-core groups in the seven genomes, suggesting high genomic flexibility. To validate this observation, we further analyzed the same genomes using anvi’o. A total of 13,111 gene clusters were identified from 29,373 genes across the seven genomes. However, in contrast to the Roary result, the anvi’o analysis identified a potential core gene category among these genomes (see Supporting data). In addition, we isolated 776 unique genes of the NS4 genome from anvi’o and analyzed their COG category distribution (Fig. S4). The most predominant functional category was “cell wall/membrane/envelope biogenesis” (14%, 45 of 321 classified genes), followed by “signal transduction mechanisms” (11%). Interestingly, Lantz et al. reported that the genome of *Ca*. Nitrotoga sp. CP45, cultivated as an acid-tolerant strain, contains numerous genes involved in cell wall and membrane biosynthesis, which may be associated with pH homeostasis (95 and references within). KEGG classification further revealed that the unique gene set of NS4 includes those involved in type IV pilus assembly, exopolysaccharide biosynthesis, prokaryotic defense systems, and baseplate protein components of bacteriophages. Especially, the genes for type IV pilus assembly and exopolysaccharide biosynthesis may have a putative role in facilitating adaptation to low pH conditions (65, 104, 105). Taken together, although this comparison involved a limited number of genomes, the results suggest that members of the genus *Nitrospira* exhibit substantial genomic plasticity and harbor distinct gene repertoires. Nevertheless, genes involved in core metabolic functions, such as carbon and nitrogen metabolism, were conserved across most *Nitrospira* genomes analyzed.

To further investigate genome plasticity, we assessed the occurrence of HGT and recombination. Although no HGT event was detected in these genomes, recombination events were predicted in all seven genomes, ranging from 7 to 31 events per genome. Three key recombination parameters were estimated: *R*/*θ* = 0.127 (0.112–0.143), *δ* = 209.54 bp (192.84–234.72 bp), and *ν* = 0.069 (0.067–0.071), corresponding to the ratio of recombination to mutation rates, the average length of recombinant fragments, and the probability of mutation within recombinant fragments, respectively. The estimated mutation rate (*θ*/*R*) was approximately 7.911. Each recombination event and the ratio of recombination to mutation were calculated to about 14.499 substitutions and 1.836, respectively (40). This estimation indicates that recombination events may generate about 1.8 times more substitutions than point mutation. These results suggest that recombination contributes more substantially to genetic variation (i.e., plasity) than point mutations and may partly explain the lack of a core gene set and the low ANI or AAI observed among the *Nitrospira* genomes (72, 106).

### Conclusions

In the (pan)genomic era, uncharacterized and uncultivated microorganisms, often referred to as “dark matter,” have been identified in various environments. Moreover, the complex diversities of microbial communities have been extensively described and deciphered. Therefore, cultivating these fastidious microorganisms is crucial for advancing our understanding. In this study, we successfully enriched the NS4 culture, a member of the genus *Nitrospira*_*D*, under acidic culture conditions and characterized its physiology, substrate kinetics, and genomic traits. Although heterotrophic microorganisms were still present in the enrichment, both *nxrB* clone library analysis and GTDB-based taxonomy supported that only a single NOB was enriched. Our findings suggest that NS4 culture is well-adapted to oligotrophic conditions with a high affinity for nitrite and is resistant to acidic environments via gene repertoire harbored in its genome. Additionally, we found that NS4 culture likely requires symbiotic partnerships (as a cobalamin provider) in its habitat. The metabolic genetic potential of NS4 genome is limited compared to other NOB, as evidenced by the absence of hydrogen metabolism and urea utilization. Collectively, NS4 culture may exhibit niche differentiation among NOB, particularly within the *Nitrospira* genus. Our findings provide insights into the potential for nitrite oxidation in acidic environments.

## Data Availability

The metagenome-assembled genome (MAG) NS4 has been deposited in DDBJ/ENA/GenBank under the accession number JBMWAN000000000. The version used in this study is JBMWAN010000000. The raw sequence data are available in NCBI under the Sequence Read Archive (SRA) with BioProject no. PRJNA1247261, and SRA no. SRR34364940 and SRR34364941. Supporting data, including MAG information (i.e., classification and completeness), genomic features, gene prediction, and functional annotations of MAG NS4, are available on FigShare (DOI: 10.6084/m9.figshare.29467067).

## SUPPLEMENTAL MATERIAL

Data Set S1 aem.01522-25-s0001.xlsx Genomic characteristics, gene prediction, and functional annotation of the MAG NS4.

Supplemental figures aem.01522-25-s0002.docx Figures S1 to S4.

Table S1 aem.01522-25-s0003.xlsx Physiological properties of cultivated representatives of the genus *Nitrospira*.

ASM does not own the copyrights to Supplemental Material that may be linked to, or accessed through, an article. The authors have granted ASM a non-exclusive, world-wide license to publish the Supplemental Material files. Please contact the corresponding author directly for reuse.
