# Supplementary material for: Genomics and physiological characterizations of an acidotolerant nitrite-oxidizing Nitrospira enriched from freshwater pond
Source: Appl Environ Microbiol. 2025 Sep 18;91(10):e01522-25. doi: 10.1128/aem.01522-25 (PMC12542776; doi:10.1128/aem.01522-25)

**Supplementary table and figure legends**

**Genomics and physiological characterizations of an acidotolerant nitrite-oxidizing *Nitrospira* enriched from freshwater pond**

Minji Kim^1^, Yoichi Kamagata^2^, and Soo-Je Park^1^*

^1^Department of Biology, Jeju National University, Jeju 63243, South Korea

^2^Bioproduction Research Institute, National Institute of Advanced Industrial Science and Technology (AIST), Tsukuba, Ibaraki, Japan.

*Corresponding author: Soo-Je Park (Tel: +82-64-753-3524, Fax: +82-64-756-3541, Email: sjpark@jejunu.ac.kr)

**Running title**: A novel acid-tolerant nitrite-oxidizing bacterium enriched from pond

**Keywords**: *Nitrospira*, Nitrite oxidation, Kinetics, Acid-tolerant, Genome, Physiology

**Table S1.** Physiological properties of cultivated representatives of the genus *Nitrospira*.

**Fig. S1.** Neighbor-joining phylogenetic tree of 16S rRNA gene sequences from the strain NS4 and close relatives (i.e., six lineage) of the genus *Nitrospira*. Phylogenetic trees were generated using MEGA 11 (<https://www.megasoftware.net/>) with 1000 bootstraps. Nodes with bootstrap support values (≥ 70%) are shown with closed circles. Bold blue denotes enriched NS4 culture in this study.

**Fig. S2.** Oxygen kinetics of the NS4 culture. Michaelis-Menten plots of oxygen uptake calculated by oxygen consumption by nitrite oxidation was shown. Experiments were performed with early-stationary-phase cultures at deﬁned incubation temperatures of 26°C and pH 6. Inner graph plotted by Lineweaver-Burk equation.

**Fig. S3.** Distribution of Clusters of Orthologous Groups (COGs) in functional classes of all genomes of *Nitrospira* spp. selected in this study. Values presented by stacked frequency (%) of genes per COG category, and x-axis represents each genome. Red boldface denotes the NS4 genome enriched in this study. The right panel shows COG functional classification.

**Fig. S4.** Frequency distribution of COG20 categories for 321 gene cluster (n=776 genes) identified in the NS4 genome using anvi'o analysis.

**Additional data**

Genomic information includes genomic characteristics, gene prediction, and functional annotation of the MAG NS4.

**Fig. S1.**


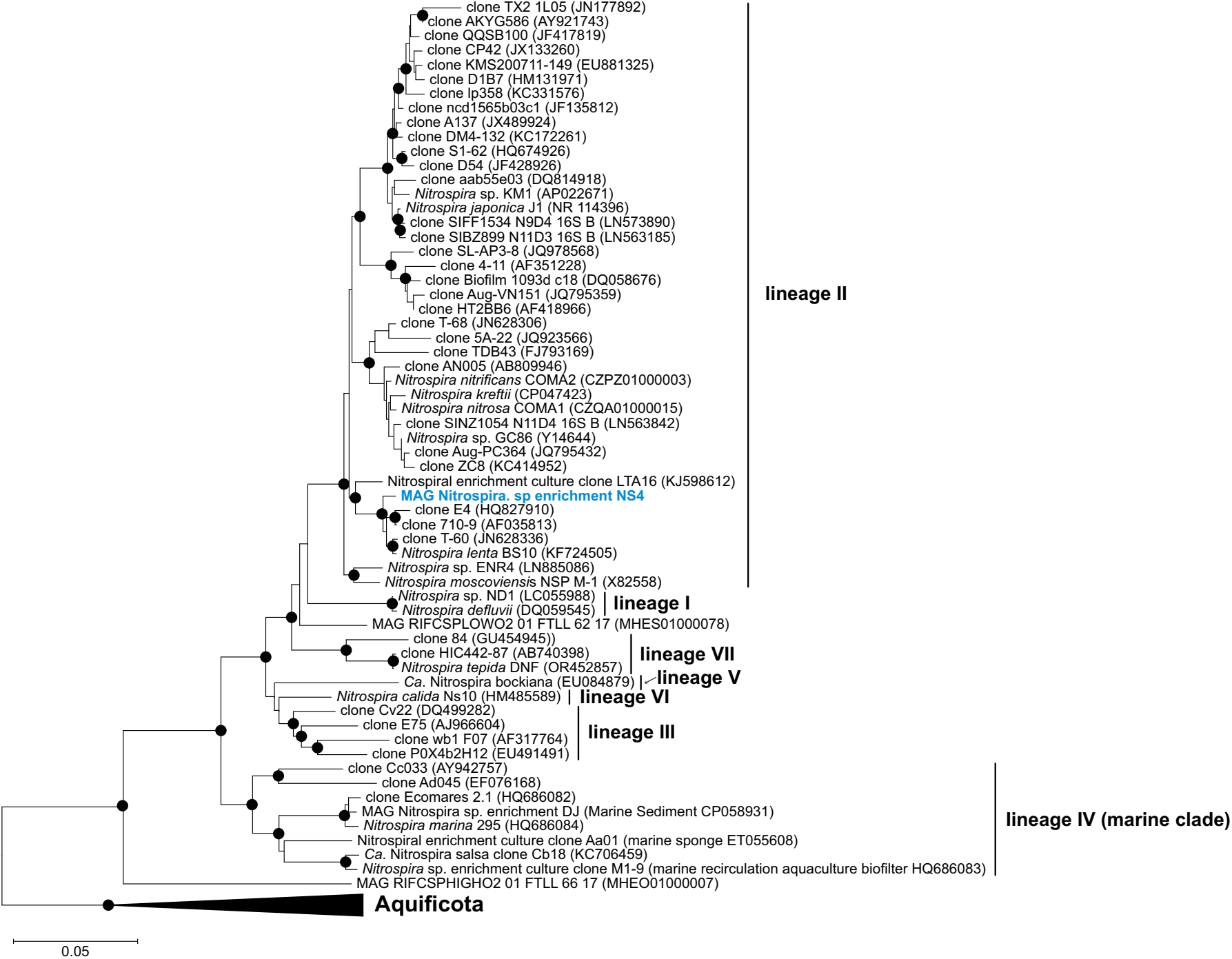


**Fig. S2.**


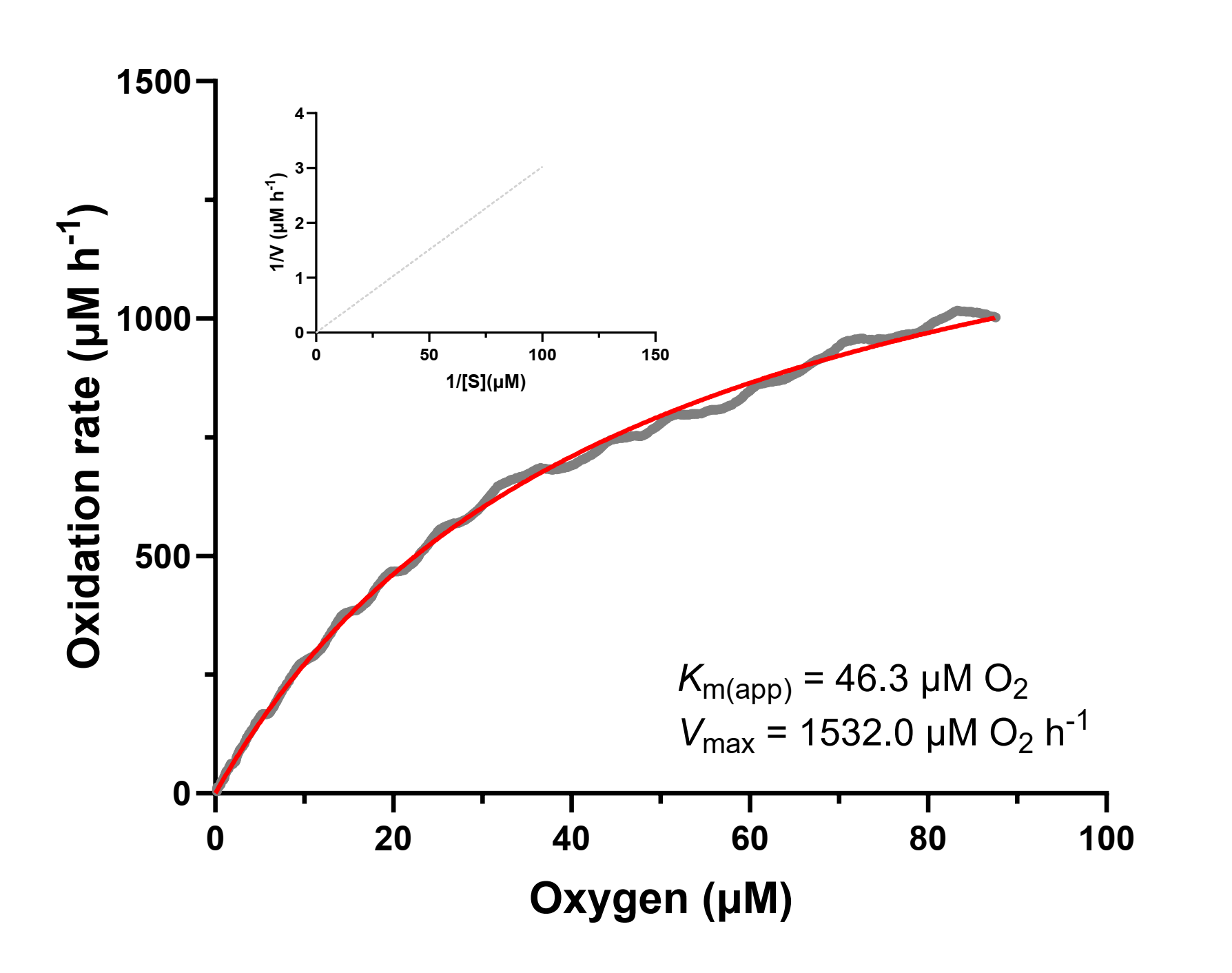


**Fig. S3.**


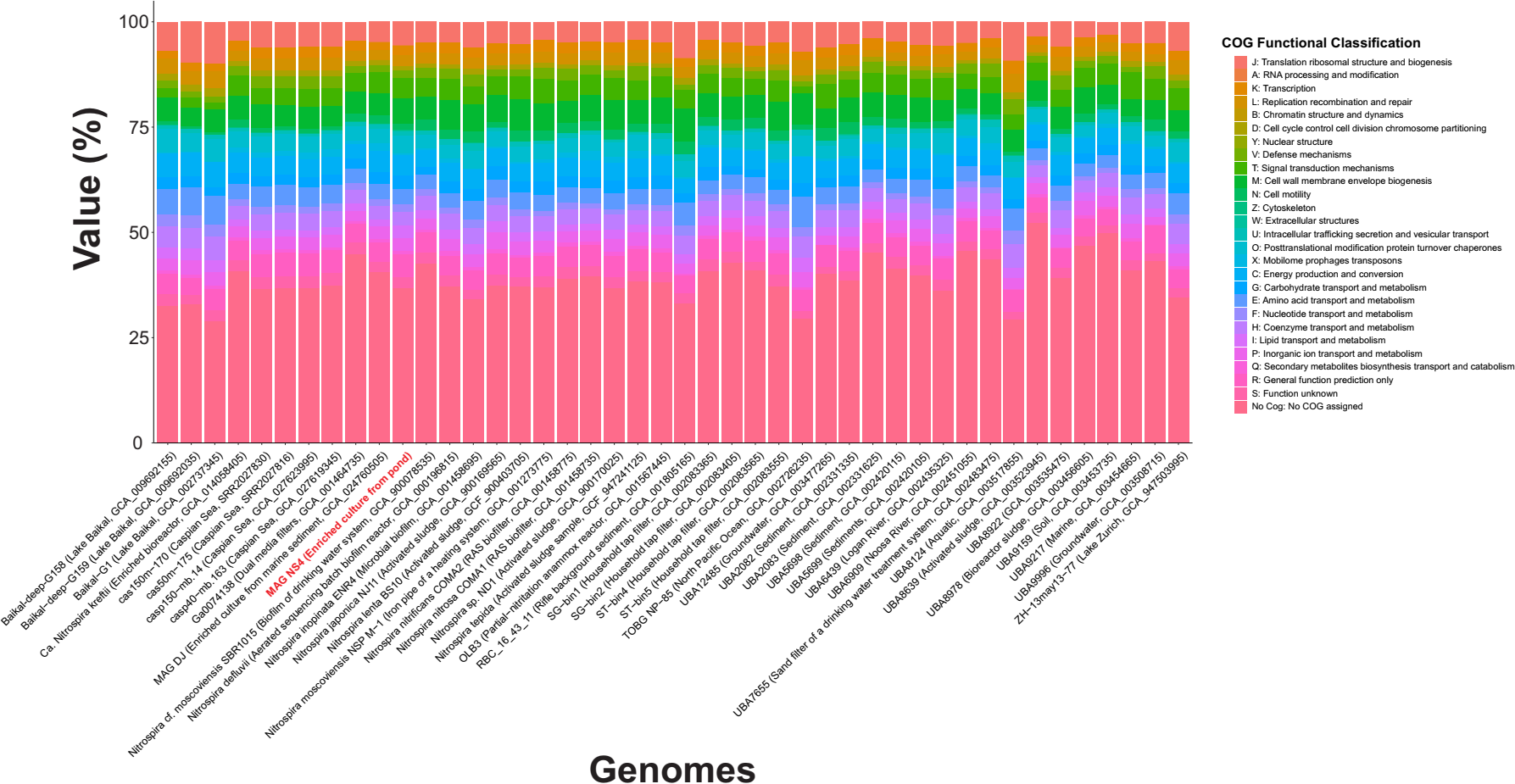


**Fig. S4.**


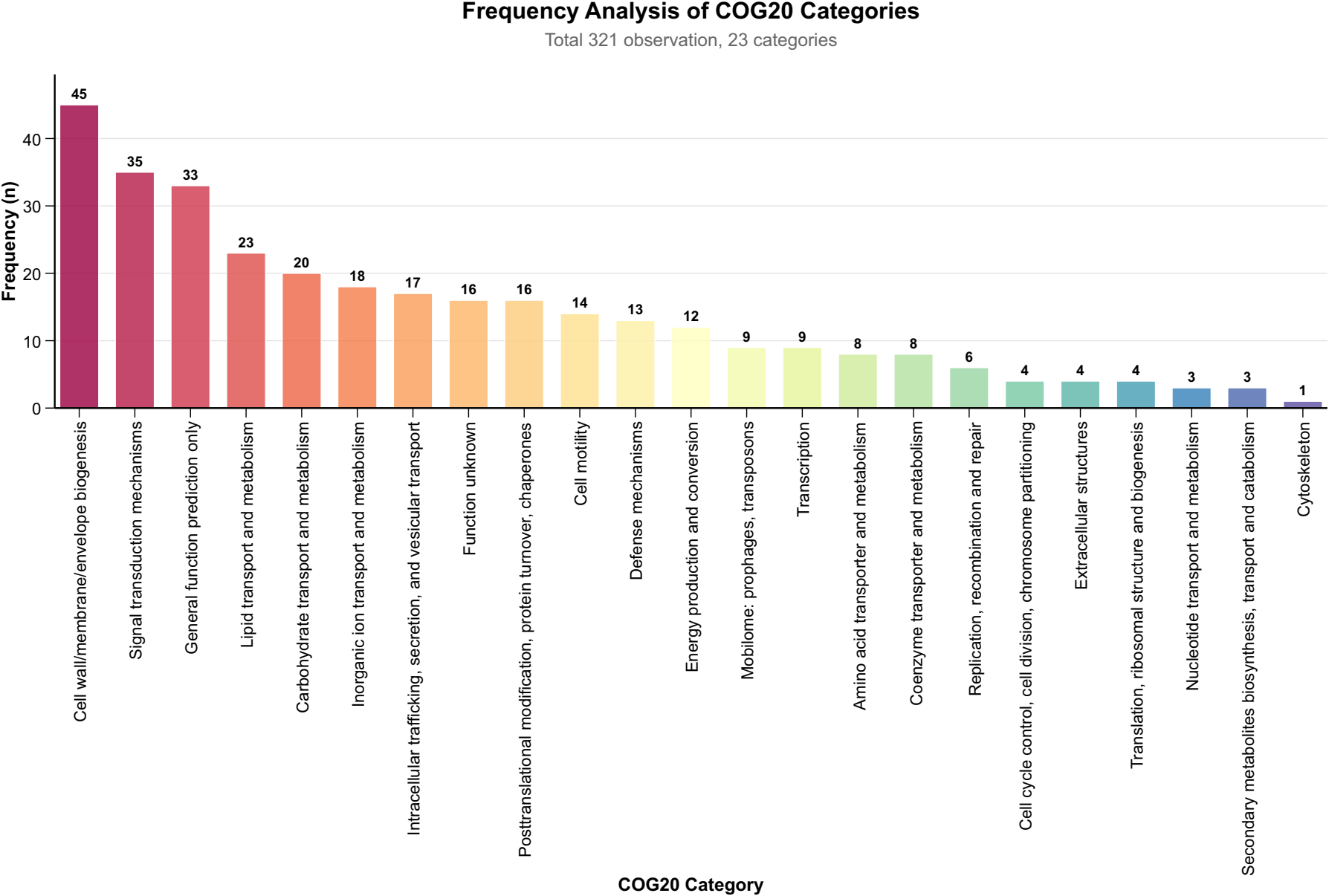

Supplement: Supplemental figures — Figures S1 to S4. [file aem.01522-25-s0002.docx]
